# MRI Verification of a 10–20 Targeting Protocol Used During Transcranial Magnetic Stimulation Sessions for Tinnitus

**DOI:** 10.1007/s10548-018-0636-9

**Published:** 2018-02-21

**Authors:** Sarah M. Theodoroff, Alexander A. Stevens, Garnett McMillan, David R. Pettersson, William Woodward, Robert L. Folmer

**Affiliations:** 1 VA RR&D National Center for Rehabilitative Auditory Research, VA Portland Health Care System, 3710 SW US Veterans Hospital Road (NCRAR – P5), Portland, OR 97239 USA; 2 Department of Otolaryngology, Head-Neck-Surgery, Oregon Health & Science University, 3181 SW Sam Jackson Park Road, Portland, OR 97239 USA; 3 Advanced Imaging Research Center, Oregon Health & Science University, 3181 SW Sam Jackson Park Road, Portland, OR 97239 USA; 4 Department of Diagnostic Radiology, Oregon Health & Science University, 3181 SW Sam Jackson Park Road, Portland, OR 97239 USA

**Keywords:** Tinnitus, Transcranial magnetic stimulation, Magnetic resonance imaging

## Abstract

Langguth et al. (2006) described a method for targeting primary auditory cortex (PAC) during transcranial magnetic stimulation (TMS) using the 10–20 electroencephalography system. Study aims were to measure the degree of accuracy in placing the TMS coil on the scalp overlying PAC using the 10–20 method and determine the extent to which accuracy depends on the hemisphere of the coil placement. Twelve participants underwent anatomical magnetic resonance imaging (MRI) of their head in a 3T scanner. Before imaging, a fiducial marker was placed on their scalp corresponding to the TMS coil position. MRI scans were analyzed to determine the distance from the fiducial marker to PAC for each participant. On average, the 10–20 method resulted in distances in the medial–lateral, anterior-posterior, and inferior-superior dimensions that were within a few millimeters (~ 4 mm) of each other between the left and right hemispheres. The fiducial marker was, on average, 10.4 mm superior and 10.8 mm posterior to the optimal scalp location that minimized the distance to PAC. Individual asymmetries and other systematic differences found in this study raise important considerations to keep in mind that might necessitate using an MRI-guided method of coil-positioning when targeting PAC for TMS.

## Introduction

Tinnitus is a phantom perception of sound in the absence of external acoustic stimulation. Although tinnitus is experienced by millions of people worldwide, the neural mechanisms giving rise to the percept remain elusive (Eggermont 2015; De Ridder et al. 2014). Tinnitus can be a debilitating condition that negatively affects quality of life and sometimes results in emotional and psychological distress, concentration problems, insomnia, and catastrophic thinking (Cima et al. 2011). To date, diagnosis of tinnitus is completely based on self-report. An individual’s emotional state contributes to how one experiences tinnitus (Krog et al. 2010). Tinnitus can only be measured indirectly and due to its multifaceted and subjective nature, exacting a “true” measure of tinnitus is difficult to achieve. The complex nature of tinnitus makes it difficult to separate the perceptual aspects (e.g., loudness, pitch) from the emotional contributions resulting in tinnitus distress; it also makes assessment of tinnitus severity challenging. This has considerable implications for evaluating evidence-based interventions and clinical trials because no widely accepted outcome measure for tinnitus exists (Landgrebe et al. 2012). Due to the lack of standardized outcome measures for tinnitus, examining and comparing the effectiveness of different tinnitus interventions across studies is problematic (Kamalski et al. 2010).

Despite these difficulties, many new treatment methods are being investigated that aim to reduce the perceived loudness, severity, and annoyance of tinnitus. Research has shown that regardless of the initial pathology associated with tinnitus (i.e., damage to peripheral auditory structures), the continued perception of tinnitus is generated by neural activity within the central auditory system (Eggermont 2003). The body of work conducted to discover the neural mechanisms underlying tinnitus has resulted in three main neurophysiological models: increased spontaneous activity of auditory neurons (Kaltenbach and Godfrey 2008), increased neural synchrony (Norena and Eggermont 2003), and tonotopic reorganization of primary auditory cortex (PAC) (Mühlnickel et al. 1998; Eggermont 2006). The underlying mechanism(s) that initially give rise to tinnitus may be different from the cascade of changes that results in tinnitus becoming chronic (Kaltenbach 2011).

Increasingly, researchers are targeting the neural systems associated with the perceptual aspects of tinnitus using transcranial magnetic stimulation (TMS). A non-invasive technique, TMS delivers electromagnetic pulses through a magnetic coil placed in contact with the patient’s scalp. Energy from the coil is transmitted through the skull inducing an electric current in underlying neural tissue and thereby affecting neuronal activity (see Theodoroff and Folmer 2013 for a history and review of TMS studies involving tinnitus patients).

Many studies have reported that patients experience reductions in tinnitus-related problems as well as the tinnitus perception itself becoming less “loud” following TMS sessions (Kleinjung et al. 2005; Langguth et al. 2006; Plewnia et al. 2007; Rossi et al. 2007; Smith et al. 2007; Khedr et al. 2008; Marcondes et al. 2010). These promising results are encouraging researchers and clinicians to explore TMS as a treatment for tinnitus. Many of these studies attempted to apply TMS to a region of the scalp overlying auditory cortex because several different neuroimaging studies have shown an association between superfluous neural activity in this region and the tinnitus percept (Arnold et al. 1996; Lockwood et al. 1998; Folmer 2007). To date, there is not wide-spread acceptance of what the most effective method or protocol is when administering TMS for tinnitus. Repetitive TMS for tinnitus treatment is still experimental and there are many procedural considerations that require further study. For example, it is still a matter of debate as to what the ideal neural target is and hemisphere side (left vs. right) for rTMS stimulation for tinnitus treatment (Theodoroff and Folmer 2013; Folmer et al. 2015). Should PAC be the optimal target, it is important to consider if this neural region can be stimulated directly using rTMS or if its activation is modulated indirectly via a more superficial path through secondary auditory cortical structures and other neural networks linked with auditory cortex such as limbic regions and frontal cortex (Romanski and Le Doux 1993; De Ridder et al. 2014; Plakke and Romanski 2014).

Taking these considerations into account, Folmer et al. (2015) followed the recommendation of Langguth et al. (2006) who developed their TMS target method for locating auditory cortex based on magnetic resonance imaging (MRI) data from a sample of 25 individuals. Using MRI data, Langguth et al. (2006) devised a method to use the International 10–20 electroencephalography (EEG) system to place the TMS coil over the skull region closest to PAC. The International 10–20 system (Jasper 1958) is the gold standard for electrode placement when performing EEG recordings. It is a systematic method of identifying standardized scalp locations that can be applied to any research participant or patient. The scalp locations are identified based on their distance (i.e., 10, 20%) between anatomical landmarks on the scalp (e.g., nasion, inion, and preaurical point).

A limitation of using a 10–20 EEG-based method is the uncertainty of whether or not specific neural regions associated with auditory cortex are actually being stimulated in individuals receiving TMS treatment. Langguth and colleagues only targeted the left hemisphere in their study. However, the perceived location of tinnitus can vary widely from person to person—it may be reported as being dominant in either ear or perceived bilaterally, suggesting that the underlying pathophysiology may also be lateralized to either hemisphere. The anatomical location of auditory cortical structures is often asymmetric in individuals (Penhune et al. 1996; Leonard et al. 1998) and therefore, it is worth assessing the accuracy of the 10–20 EEG-based method for positioning the TMS coil when targeting the right PAC as well as the left.

Differences in brain anatomy have been reported in people of different races. This fact motivated Noh et al. (2017) to conduct a study in Korea investigating the location accuracy of Langguth et al’s 10–20 approach to target PAC in Asian individuals. Noh et al. were particularly interested in how differences between Asian and Caucasian skull dimensions might affect the accuracy of Langguth et al’s 10–20 approach for targeting PAC; they used anatomical MRI in 17 Asian participants to answer their research question and calculated the relative difference between the optimal scalp target (determined by neural imaging) and the scalp target determined by Langguth’s 10–20 EEG technique.

This study was conducted to assess the accuracy of the 10–20 EEG-based approach in targeting auditory cortical structures in both hemispheres used in the Folmer et al. (2015) clinical trial whose vast majority of participants were Caucasians from the United States of America. Addressing coil placement accuracy is important because of the mechanisms by which TMS affects neural activity. With each TMS pulse, energy from the TMS coil is transmitted through the scalp and induces an electric current in underlying neural tissue; therefore, it is crucial to place the TMS coil on the scalp in a location that provides the most direct path to underlying neural structures of interest. The farther the TMS coil is placed on the scalp from underlying neural tissue and structures, the less likely it is that the electromagnetic field generated by TMS will affect the intended neural targets.

The aims of this study were to: (1) measure the degree of accuracy in placing the TMS coil on the scalp overlying PAC (i.e., Heschl’s gyrus) using Langguth et al’s 10–20 approach, and (2) determine the extent to which TMS coil placement accuracy depends on the side of the head (i.e., left vs. right). These research questions were addressed by evaluating: (I) the Euclidian distance between the TMS coil on the scalp and PAC, a measure that yields information about the relative distance from the location of the TMS coil placed on the scalp to the neural target (in the medial–lateral plane); and (II) the distance between the TMS coil on the scalp and the optimal scalp location for placement of the TMS coil, a measure that yields information about the relative distance between the actual location of the TMS coil on the scalp compared to the optimal placement (in the inferior-superior and anterior-posterior planes). Specifically, the scalp distance measure (II) will address our research question about the degree of accuracy of Langguth et al.’s (2006) 10–20 approach to target PAC in our sample.

## Materials and Methods

### Participant Information

Twelve individuals (5 males; 7 females) who ranged in age from 35 to 74 years (mean = 62 years; SD = 10 years) participated in this study. These individuals all participated in the Folmer et al. (2015) clinical trial and were recruited for the current study.

The VA Portland Health Care System’s Institutional Review Board and Oregon Health & Science University’s Institutional Review Board reviewed and approved the current study’s protocol. All participants provided informed consent prior to any procedures being performed in accordance with the ethical standards addressed in the 1964 Declaration of Helsinki.

### Magnetic Resonance Imaging

Prior to imaging being performed, all participants completed an MRI screening questionnaire to verify it was safe for them to undergo this procedure. All magnetic resonance (MR) scans were acquired on a Siemens 3 T Tim-Trio system (Siemens, Erlengen, Germany) fitted with a 12-channel parallel array headcoil located in the Advanced Imaging Research Center at Oregon Health & Science University, in Portland Oregon. Prior to MR scanning, a small fiducial marker (Multi-Modality Radiology Marker, IZI Medical Products, Owings Mills, MD) was attached to the participant’s scalp at the target location according to the 10–20 EEG-based positioning method described by Langguth et al. (2006). The fiducial marker was placed on the participants’ scalp corresponding to the TMS target coil position (left or right hemisphere) associated with where they received rTMS during the clinical trial (Folmer et al. 2015).

To localize the anatomical structures associated with human auditory cortical regions (e.g., transverse temporal gyri, planum temporale, superior temporal gyrus, superior temporal sulcus), structural images were obtained using a sagittal magnetization-prepared rapid gradient echo (MP-RAGE) three-dimensional T1-weighted sequence (TR = 9.7 ms, TE = 4 ms, flip angle = 12°, TI = 300 ms, voxel size = 1.25 × 1 × 1 mm, slices = 128). All scans were reviewed by a neuroradiologist (David R. Pettersson, M.D.) to screen for neuropathology.

### MRI Data Analysis

Anatomical datasets were processed for reconstruction using the Freesurfer software toolkit (http://freesurfer.net) and Analysis of Functional Neuroimages (AFNI) programs (Cox 1996). Data were submitted to the Freesurfer “recon-all” function, using default settings. A full description of the processing steps is provided online (https://surfer.nmr.mgh.harvard.edu/fswiki). Briefly, each brain dataset was intensity normalized and registered to a standard (Talairach) atlas space. This included alignment to the anterior commissure-posterior commissure plane, and along the mid-sagittal plane. The brain was then automatically parcellated into cortical regions, including the PAC, using the Desikian atlas within Freesurfer (Fischl et al. 2004; Desikan et al. 2006). The brains and the PAC mask were then converted back into individual brain space to avoid introducing spatial distortions while preserving the standardized multi-plane alignment.

### Operational Definitions

#### Definition of PAC

**Fig. 1:**
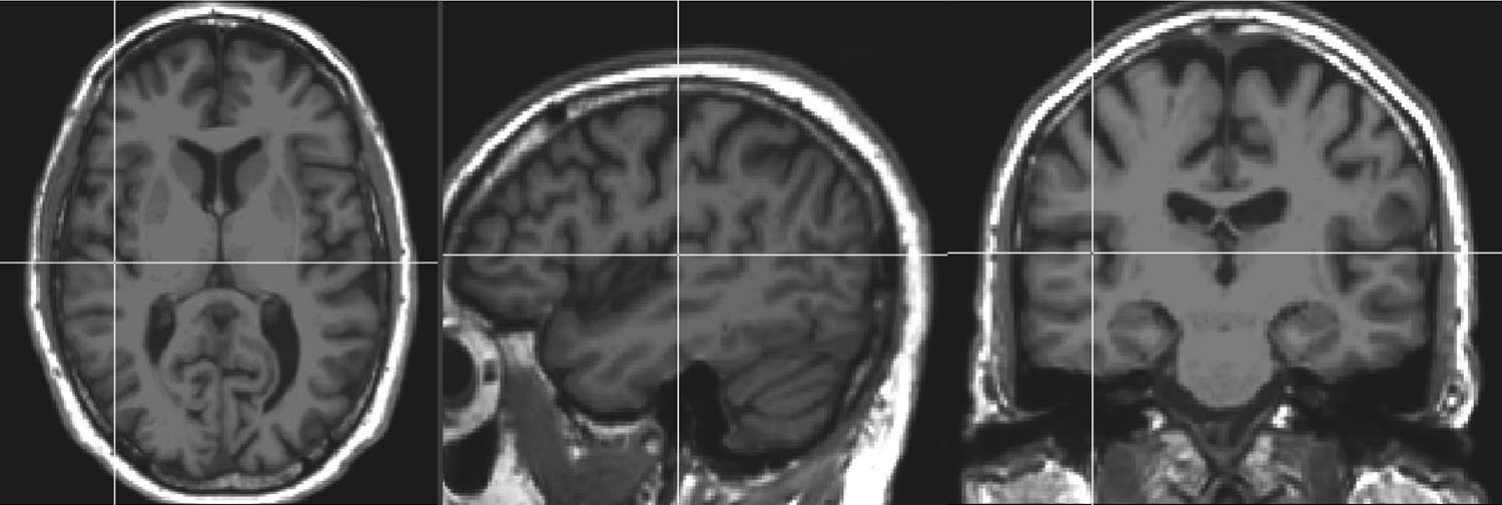
Example of the center of primary auditory cortex (crosshairs) determined by measuring the trunk and center of mass of the transverse temporal gyrus (see text for details)

Typically, PAC occupies the medial two-thirds of the transverse temporal gyrus (TTG), and in cases where there are two branches of the TTG, it resides on the anterior branch (Liegeois-Chauvel et al. 1991; Leonard et al. 1998). We used the center of the PAC as a standardized reference point, based on the description provided in Langguth et al. (2006). The exact coordinates of the voxel identified as the anatomical center of the PAC was identified as follows: the gray matter mask of the TTG from the Desikan atlas in Freesurfer was applied to each individual brain and the midpoint between the most anterior and posterior voxels along the medial border of the TTG was located. Next, we computed the center of mass of the gray matter mask of the TTG, and projected a line along the axis of the trunk of the TTG through the Center of Mass of the TTG to its lateral surface. The point located at 1/3 the length of the axis from the medial end of the TTG was used as the reference point for the PAC. Figure 1 shows an example of the center of the PAC using this measurement technique. All analyses of distances used this as the PAC reference point.

### I. Distance Between TMS Coil on the Scalp and PAC

#### Optimal Distance: Measuring the Shortest Distance Between the Scalp and PAC

To find the shortest linear path from PAC to the scalp, a mask was first made of the scalp and merged with the brain mask, setting all voxels external to the scalp to zero. A sphere was placed with its origin at the coordinate of the PAC in the same hemisphere as the fiducial marker, and progressively expanded until it intersected the surface of the scalp mask. The Euclidean distance from PAC was calculated for all voxels in the scalp surface mask intersecting the sphere, sorted by path length. The voxel identified with the shortest distance from PAC was confirmed to be located on the scalp by visual inspection.

#### Fiducial Distance: Measuring the Realized Distance Between the Fiducial Marker Placed on the Scalp (Where the TMS Coil Was Placed on the Scalp) and PAC

The distance between the location of the fiducial marker on the scalp and the brain coordinate space of the PAC was calculated for each plane within the three-dimensional brain coordinate space, taking the difference between the coordinates associated with each orthogonal plane (medial–lateral, anterior-posterior, and the inferior-superior plane):

$$distance= \sqrt{{({x}_{fid}-{x}_{PAC})}^{2}+{({y}_{fid}-{y}_{PAC})}^{2}+{({z}_{fid}-{z}_{PAC})}^{2}}$$

#### Defining Accuracy

Degree of accuracy was defined as the distance between PAC and the scalp position according to the fiducial marker placement or by the calculated optimal placement. Relative accuracy is defined as the difference between the fiducial distance and optimal distance from the scalp to the PAC and from the fiducial scalp location to the optimal scalp location. In the current study, optimal placement refers only to the position that minimizes the distance from the scalp to PAC; the extent to which PAC is stimulated by TMS is unknown.

#### Statistical Analysis

For each participant, the distances from the fiducial marker to the PAC reference point and the optimal scalp location in the same hemisphere were measured in all three planes (medial–lateral, anterior-posterior, and inferior-superior) and the Euclidian distance was calculated.

### II. Distance Between Actual TMS Coil on the Scalp and the Optimal TMS Coil Scalp Location

To estimate the offset of the 10–20 EEG-defined TMS scalp location to the optimal TMS scalp location for each participant, the relative distance between the placement of the TMS coil on the scalp and the optimal placement for the coil in both hemispheres was calculated in the inferior-superior and anterior-posterior planes.

## Results

The results from the two types of measurements described above: (I) distance between TMS coil on the scalp and PAC; and (II) distance between where the TMS coil was placed on the scalp compared to the optimal placement are labeled with “I” or “II” accordingly.

I. Table 1 displays the mean fiducial distance and optimal distance to the PAC across all participants in the medial–lateral, anterior-posterior, and inferior-superior planes for both left and right hemispheres. The greatest distance between the fiducial and the PAC in any plane occurred in the medial–lateral direction, reflecting the limit imposed by the scalp and skull, and the relatively deep location of the PAC.

**Table 1:** Means and standard deviations of distance (in millimeters) between the fiducial marker and the target within primary auditory cortex (PAC) and the optimal distance to PAC for each hemisphere in the medial–lateral, anterior-posterior, and inferior-superior brain coordinate space

Hemisphere	Medial–lateral	Anterior–posterior	Inferior–superior	Distance to PAC
Fiducial distance				
Left	31.8 (3.0)	11.0 (7.6)	15.4 (5.4)	37.9
Right	35.6 (4.2)	10.2 (7.4)	11.2 (4.8)	39.5
Optimal distance				
Left	32.9 (2.9)	2.6 (2.4)	5.5 (2.9)	33.7
Right	35.0 (3.8)	7.6 (2.8)	4.7 (3.9)	36.3

The medial–lateral data in Table 2 represents the difference in the distance from the fiducial marker location to PAC and the optimal scalp location to PAC. These results indicate that the optimal scalp location was, on average, 3.8 mm nearer to PAC (SD = 1.5) compared to the fiducial scalp location.

**Table 2:** Difference between the fiducial marker and the optimal scalp location for each hemisphere in the medial–lateral, anterior-posterior, and inferior-superior dimensions. Mean and standard deviations (SD) are provided in millimeters (mm)

Hemisphere	Difference between fiducial and optimal scalp location (mm)		
	Medial–lateral	Anterior–posterior	Inferior–superior
Left	6.6	22—posterior	15—superior
Right	4.4	24—posterior	2—superior
Left	4.4	4—anterior	15—superior
Left	4.8	20—posterior	8—superior
Right	1.4	3—anterior	11—superior
Left	3.5	16—posterior	18—superior
Left	3.4	8—posterior	12—superior
Left	3.2	16—posterior	10—superior
Left	4.0	6—anterior	14—superior
Right	6.0	23—posterior	9—superior
Right	2.3	21—posterior	6—superior
Right	1.7	8—anterior	5—superior
Mean (SD)	3.8 (1.5)	10.8 (12.3)	10.4 (4.6)

**Fig. 2:**
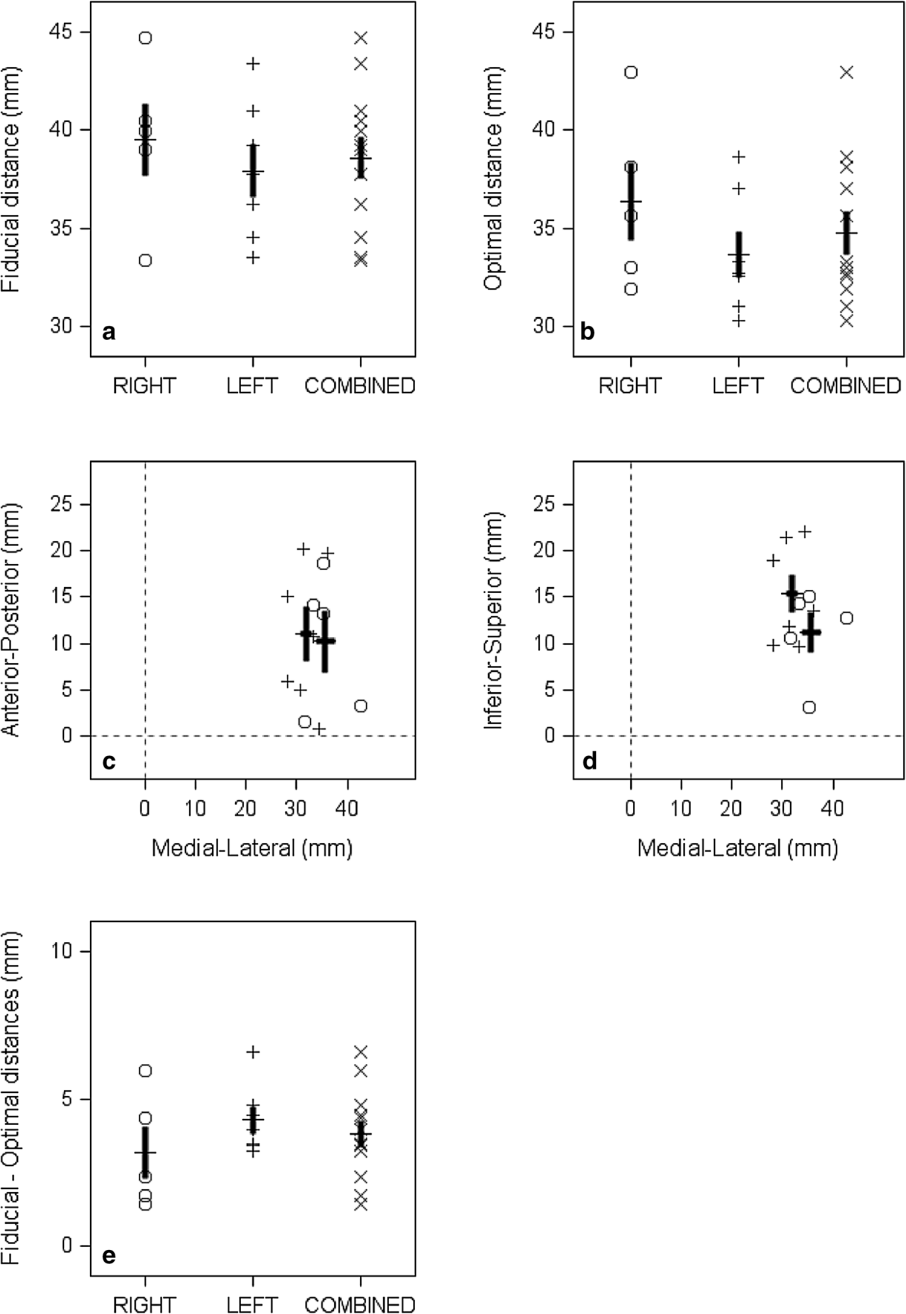
The **a** Euclidian distance between the fiducial marker and the center of primary auditory cortex (PAC). These data are sorted by the hemisphere of fiducial placement for each participant and the cross designates the mean value; **b** the Euclidian distance to the nearest scalp location from the center of PAC as a function of hemisphere placement; **c** the distance of the fiducial from the PAC in the anterior-posterior and **d** inferior-superior planes as a function of the medial–lateral plane. The crosshairs are the mean distance (± 1 standard error) of the fiducial marker in each hemisphere. The dotted lines cross at the center of the PAC, which is used as the origin; **e** the overall difference between the fiducial marker scalp distance and the optimum minimum distance in placing the TMS coil over PAC as a function of right or left hemisphere placement or combined (left and right hemispheres)

Individual results displaying the Euclidian distance between the fiducial marker and the center of PAC are displayed in Fig. 2a. The optimal distance between the scalp and PAC are displayed in Fig. 2b and represent the Euclidean distance to the nearest scalp location from the center of PAC for left vs. right hemisphere. Next, we calculated whether the 10–20 EEG fiducial distance measurements were comparable in the medial–lateral, anterior-posterior and inferior-superior planes across both hemispheres for each participant (Fig. 2c, d). The intersection of the dotted lines in Fig. 2c, d indicate the center of PAC which is set at the origin (0,0) of the graph. The distances between the origin and each data point indicate the fiducial distance for each participant. The centers of the crosshairs (dark solid black lines) indicate the mean distance of the participants’ fiducial marker placement from PAC, with one standard error above and below the mean indicated by length of the crosshairs.

In all cases, the fiducial marker was placed lateral to the PAC, which reflects the simple fact that the skull imposes a limit on the minimum lateral distance of the fiducial to the PAC and that the temporal scalp region overlying one hemisphere is always lateral to PAC on that side. Results show that the position of the coil sometimes resulted in fiducial marker placement superior to the axial plane of the PAC (Fig. 2c), and other times posterior to the coronal plane of the PAC (Fig. 2d). The difference between the fiducial distance and optimal distance for the combined data set as well as separated by hemisphere are displayed in Fig. 2e. To examine any possible difference between degree of measurement accuracy in placing the fiducial marker according to the 10–20 EEG-based method for the left and right hemispheres, we calculated the Bayesian confidence intervals which showed an approximate 1 mm difference in accuracy (95% confidence intervals of − 0.8 to 3.2) in favor of the right hemisphere.

II. Table 2 shows the individual and mean differences (in millimeters) between the fiducial marker on the scalp and the optimal scalp location for each dimension and hemisphere. The anterior-posterior and inferior-superior data in Table 2 compare our fiducial scalp location (determined by the 10–20 EEG-based method recommended by Langguth et al. 2006) to the optimal scalp location (shortest distance from the scalp to PAC) as determined by neural imaging. Of the 12 study participants, the fiducial marker was placed superior to the optimal scalp location in all cases, and posterior to the optimal scalp location in 7 participants. On average, the fiducial scalp location was 10.8 mm posterior (standard deviation (SD) = 12.3) to the optimal scalp location and 10.4 mm superior (SD = 4.6) to the optimal scalp location.

**Fig. 3:**
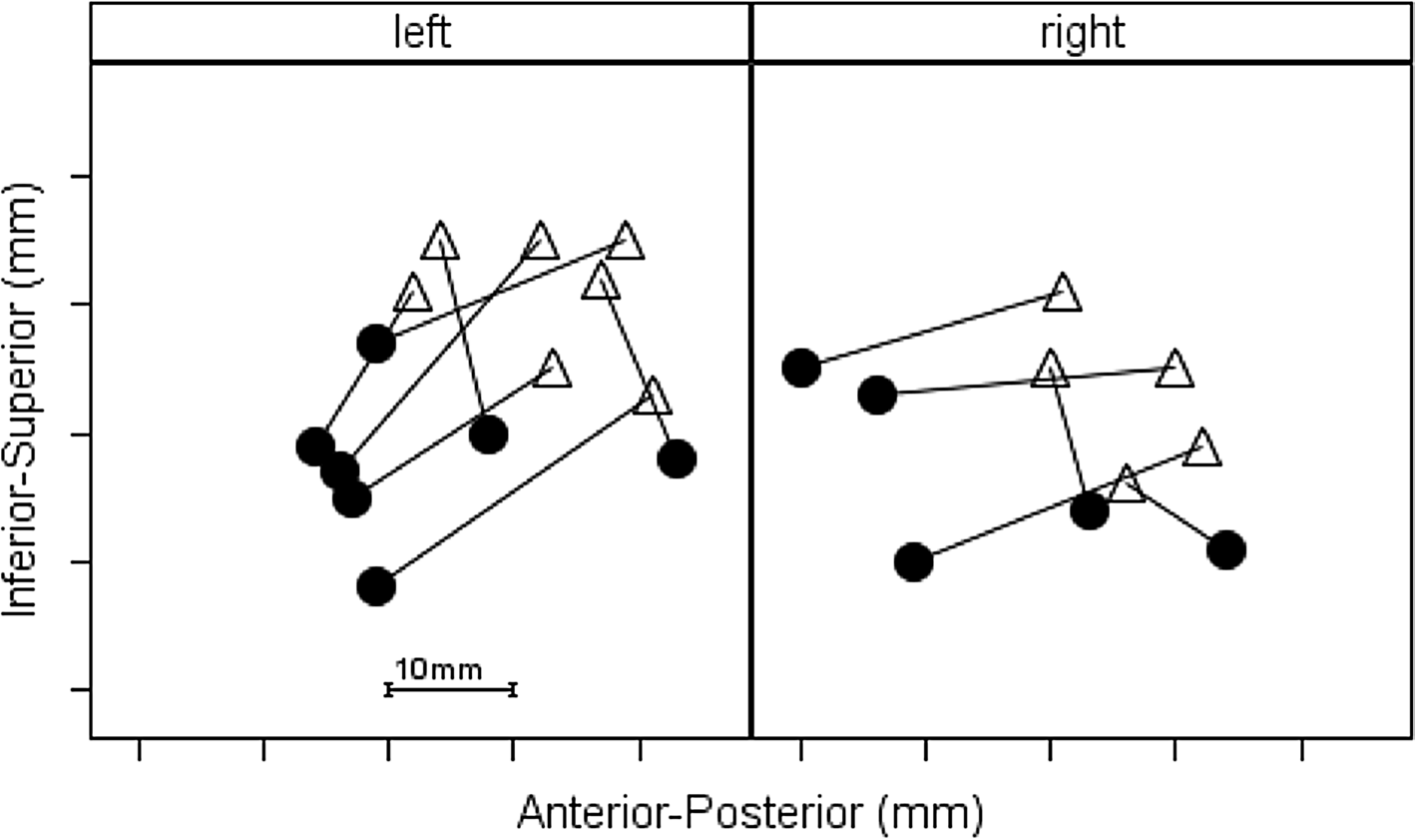
Scalp location of fiducial marker (open triangle) compared to optimal coil location (filled circle) in both hemispheres and in two dimensions: Inferior-Superior and Anterior-Posterior in millimeters (mm)

Figure 3 displays the relative distance between the fiducial scalp location and the optimal scalp location in both hemispheres in two dimensions: inferior-superior and anterior-posterior. In all 12 participants, the 10–20 EEG-based method resulted in fiducial marker placement superior to the optimal scalp location. Also, the majority of fiducial markers (8 of 12) were placed posterior to the optimal scalp location.

While it appears that there is little difference between the degree of measurement accuracy in the two hemispheres using the 10–20 EEG-based method, it is notable that the right hemisphere fiducial distance in the inferior-superior axis was approximately 4 mm lower than in the left hemisphere (Table 1; Fig. 2c,d). This may be important because that difference in the superior position would, in some cases, place the coil above the lateral sulcus. Table 3 shows the neural structures closest to the TMS coil/fiducial marker for each of the 12 participants, and includes whether the coil position fell above or below the lateral sulcus. In six cases, the coil placement was above the lateral sulcus, one was below, and five were aligned with lateral sulcus.

**Table 3:** Cortical areas underlying the fiducial marker for each participant and if the fiducial marker was above, below, or on the lateral sulcus

Hemisphere	Nearest ROI	Lateral sulcus (LS)
L	Post central gyrus	Above
L	Inferior parietal gyrus (IPG)	Above
L	Inferior parietal gyrus, planum temporale	On LS
L	Inferior parietal gyrus	Above
L	Inferior parietal gyrus	Above
L	Post central gyrus	Above
L	Planum temporale	On LS
R	Inferior parietal gyrus	On LS
R	Inferior parietal gyrus	Above
R	Superior temporal gyrus (STG) lateral	Below
R	Inferior parietal, superior temporal gyrus lateral	On LS
R	IPG/Superior medial gyrus, STG lateral	On LS

## Discussion

The main aim of this study was to determine the degree of accuracy of the 10–20 EEG-based method described by Langguth et al. (2006) for positioning the TMS coil over PAC for both the left and right hemispheres. Langguth et al. proposed a 10–20 method to locate a scalp target for the TMS coil based on MRI data from 25 individuals. Their optimal coil position was determined by calculating the average distance between the participant’s scalp and PAC. This corresponds to our measure of the realized distance between the fiducial marker placed on the scalp and PAC. By using the approach prescribed by Langguth et al., our results revealed the Euclidean distance between the scalp fiducial marker and PAC were similar in both hemispheres; however, like the results reported by Langguth et al., there was substantial inter-participant variability in the location of the fiducial marker’s placement in the three anatomical planes. Furthermore, there are systematic differences in the location of the fiducial marker relative to the PAC in the current study population compared to results reported by Langguth et al.

Our results support the findings of Noh et al. (2017) who revealed that Langguth et al’s targeting method produced systematic differences in their respective sample of Asian individuals, suggesting that the 10–20 EEG-based approach may have limited generalizability. Specifically, Noh et al’s results indicated that the scalp location identified by Langguth’s 10–20 EEG-based method was approximately 9 mm posterior and 7 mm superior to the optimal scalp location identified by MR imaging. Noh et al. attributed these variations to anatomical differences between Asian and Caucasian skull shape/size in the two study populations.

In our study of 12 North American Caucasian individuals, the scalp location identified by Langguth’s method was also posterior (mean = 10.8 mm, SD = 12.3) and superior (mean = 10.4 mm, SD = 4.6) to the optimal scalp location identified by MR imaging. Since the optimal scalp location for targeting PAC in our study was quite similar to the optimal location reported by Noh et al., differences in Asian and Caucasian head shape/size might not be the primary reason that Noh’s results differed from Langguth’s. Instead, different methods used for measuring, calculating, and mapping the 10–20 EEG coordinates might have contributed to different results obtained in these three studies.

Our data showed anatomical differences between the left and right TTG, consistent with the findings of Penhune et al. (1996) and Leonard et al. (1998) that also described the asymmetry of auditory cortical structures, not only between participants, but also between the left and right hemispheres within an individual. Results showed that the left TTG is angled in a more anterior orientation than the right TTG. This difference may be important to consider when positioning the TMS coil over this region.

The position of the fiducial marker in the inferior-superior direction ranged between 3 and 23 mm superior to PAC, with the right hemisphere position somewhat closer to the neural target (Fig. 2d). The inferior-superior position of the TMS coil is important because more superior positioning could place the coil above the lateral sulcus which may substantially alter stimulation of PAC and associated cortical structures. Examining combined left and right hemisphere data, on average, there was a 3–5 mm difference between the fiducial distance and optimal distance to PAC (Fig. 2e). In terms of relative accuracy, our findings suggest that Langguth’s 10–20 EEG-based method allows for targeting of PAC within 10–11 mm of the optimal scalp location. There is no universally agreed upon standard for defining rTMS accuracy. There are multiple sources of variability to consider, all of which could contribute to reduced accuracy for this method. For example, the 10–20 EEG-based method is open to interpretation regarding finding specific locations (e.g., preaurical point, Cz, T3, C3). Also, Langguth et al. (2006) recommended a scalp target based on average data from a relatively small sample. Given the results reported by Noh et al. (2017) and the current study, individual anatomical differences may play too large of a role to allow for a TMS target based on group averages to be used.

The clinical trial by Folmer et al. (2015) was one of few studies that delivered rTMS to either the left or right side of the head—participants were randomized into one group or the other. Because the asymmetry of auditory cortical structures is well established, it was important to examine and estimate the accuracy of using the method proposed by Langguth et al. (2006) when placing the TMS coil over the right side of the head as well as the left side. Figure 3 displays the results in separate panels for the left vs. right hemisphere and shows that the fiducial marker was most often placed superior and posterior to the optimal scalp location regardless of hemisphere.

In both the current study and Langguth et al. study (2006), the majority of participants were female (7 of 12 and 16 of 25 respectively). By contrast, the majority of participants in Noh et al (2017) study were male. Typically, females have a smaller head circumference and also exhibit differences in cortical anatomy compared to males, which are important variables to consider when using average-based measurements. In spite of inter-participant variability, our results show that the TMS coil placement for most study participants was relatively close to the target proposed by Langguth et al. (2006).

Using the 10–20 EEG-based method to position the TMS coil for tinnitus research or treatment has both benefits and drawbacks compared to MRI-based targeting. A clear benefit is that the 10–20 EEG-based method is considerably less expensive and less time-consuming than MRI data acquisition. Additionally, individuals who cannot undergo MR imaging due to exclusionary factors (e.g., implanted devices, claustrophobia, weight, etc.) would be excluded from MRI-guided rTMS studies/treatment. However, the 10–20 EEG based method cannot provide the same level of individual accuracy as MRI-based coil positioning. Consequently, using the 10–20 EEG-based method for positioning the TMS coil may be ineffectual for targeting the PAC in some individuals.

Another consideration is that PAC is located medially and tangentially to the scalp surface, raising questions about how effectively rTMS can stimulate the PAC (Bijsterbosch et al. 2012). For example, the average distance from the scalp fiducial marker to PAC was between 35 and 40 mm in this study (see Table 1), which is near the penetration limit for rTMS intensities used in the Folmer et al. clinical trial (2015). Furthermore, stimulation occurs in tissue that is orthogonal to the magnetic field and drops off substantially in the sulci that run parallel to it (Thielscher et al. 2011; Opitz et al. 2014). In most rTMS studies that target PAC, the cortical areas receiving the greatest levels of TMS energy are likely the lateral surface of the superior temporal gyrus and the inferior ends of the precentral and postcentral gyri where they abut to the temporal lobe. Based on results of this imaging study, some participants in the Folmer et al. clinical trial (2015) also received stimulation in the region of the lateral planum temporale and inferior parietal lobule. These results suggest that rTMS applied according to the 10–20 EEG-based method targeting PAC may modulate activation of neural pathways that exist from lateral auditory cortex to limbic regions and frontal cortex (Romanski and Le Doux 1993; Romanski et al. 1999; Schecklmann et al. 2013; De Ridder et al. 2014; Plakke and Romanski 2014; Carpenter-Thompson et al. 2015). In summary, rTMS stimulation targeting PAC probably has a greater effect on more lateral cortical structures than on PAC itself.

A final consideration in using the 10–20 EEG-based method to position the TMS coil is possible measurement error associated with this procedure. Errors can be made when measuring by hand the distance between anatomical landmarks on the scalp (e.g., nasion to inion). Any miscalculations in determining Cz, T3, or C3 will result in miscalculations of the rTMS target location. It is possible that variations in scalp measurement methods contributed to differences in rTMS target locations in this study compared to those reported by Langguth et al. (2006) and Noh et al. (2017). One way to address this issue is to use a neuro-navigational positioning system for TMS coil placement.

## Conclusions

To date, evidence is insufficient to state conclusively what the best neural target region(s) for rTMS treatment of tinnitus might be. This study examined right-sided as well as left-sided accuracy using the 10–20 EEG-based method proposed by Langguth et al. (2006) to position the TMS coil over PAC. Overall, the 10–20 EEG-based method defined a TMS coil scalp location that was 10.4 mm superior and 10.8 mm posterior to the optimal scalp location for targeting PAC. Individual asymmetries in auditory cortex and systematic differences found between this study and Langguth et al. (2006) indicate that an MRI-guided method of positioning the TMS coil when administering this intervention is preferable to the 10–20 EEG-based method of target determination.

## Abbreviations

AFNI: Analysis of functional neuroimages
EEG: Electroencephalography
MRI: Magnetic resonance imaging
NCRAR: National Center for Rehabilitative Auditory Research
PAC: Primary auditory cortex
SD: Standard deviation
rTMS: Repetitive transcranial magnetic stimulation
TMS: Transcranial magnetic stimulation
TTG: Transverse temporal gyrus

## References

[CR1] Arnold W, Bartenstein P, Oestreicher E, Römer W, Schwaiger M (1996). Focal metabolic activation in the predominant left auditory cortex in patients suffering from tinnitus: a PET study with [18F] deoxyglucose. ORL.

[CR2] Bijsterbosch JD, Barker AT, Lee KH, Woodruff PWR (2012). Where does transcranial magnetic stimulation (TMS) stimulate? Modelling of induced field maps for some common cortical and cerebellar targets. Med Biol Eng Comput.

[CR3] Carpenter-Thompson JR, Schmidt S, McAuley E, Husain FT (2015). Increased frontal response may underlie decreased tinnitus severity. PLoS ONE.

[CR4] Cima RF, Crombez G, Vlaeyen JW (2011). Catastrophizing and fear of tinnitus predict quality of life in patients with chronic tinnitus. Ear Hear.

[CR5] Cox RW (1996). AFNI: Software for analysis and visualization of functional magnetic resonance neuroimages. Comput Biomed Res.

[CR6] De Ridder D, Vanneste S, Weisz N (2014). An integrative model of auditory phantom perception: tinnitus as a unified percept of interacting separable subnetworks. Neurosci Biobehav Rev.

[CR7] Desikan RS, Ségonne F, Fischl B (2006). An automated labeling system for subdividing the human cerebral cortex on MRI scans into gyral based regions of interest. NeuroImage.

[CR8] Eggermont JJ (2003). Central tinnitus. Auris Nasus Larynx.

[CR9] Eggermont JJ (2006). Cortical tonotopic map reorganization and its implications for treatment of tinnitus. Acta Otolaryngol Suppl.

[CR10] Eggermont JJ (2015). Tinnitus and neural plasticity (Tonndorf lecture at XIth International Tinnitus Seminar., Berlin, 2014). Hear Res.

[CR11] Fischl B, van der Kouwe A, Destrieux C (2004). Automatically parcellating the human cerebral cortex. Cereb Cortex.

[CR12] Folmer RL (2007). Lateralization of neural activity associated with tinnitus. Neuroradiology.

[CR13] Folmer RL, Theodoroff SM, Casiana L, Shi Y, Griest S, Vachhani J (2015). Repetitive transcranial magnetic stimulation (rTMS) treatment for chronic tinnitus: results of a randomized, placebo-controlled clinical trial. JAMA Otolaryngol Head Neck Surg.

[CR14] Jasper H (1958). Report of the committee on methods of clinical examination in electroencephalography. Appendix: the twenty electrode system of the International Federation. Electroenceph clin Neurophysiol.

[CR15] Kaltenbach JA (2011). Tinnitus: models and mechanisms. Hear Res.

[CR16] Kaltenbach JA, Godfrey DA (2008). Dorsal cochlear nucleus hyperactivity and tinnitus: are they related?. Am J Audiol.

[CR17] Kamalski DM, Hoekstra CE, van Zanten BG, Grolman W, Rovers MM (2010). Measuring disease-specific health-related quality of life to evaluate treatment outcomes in tinnitus patients: a systematic review. Otolaryngol Head Neck Surg.

[CR18] Khedr EM, Rothwell JC, Ahmed MA, El-Atar A (2008). Effect of daily repetitive transcranial magnetic stimulation for treatment of tinnitus: comparison of different stimulus frequencies. J Neurol Neurosurg Psychiatry.

[CR19] Kleinjung T, Eichhammer P, Langguth B (2005). Long-term effects of repetitive transcranial magnetic stimulation (rTMS) in patients with chronic tinnitus. Otolaryngol Head Neck Surg.

[CR20] Krog NH, Engdahl B, Tambs K (2010). The association between tinnitus and mental health in a general population sample: results from the HUNT study. J Psychosom Res.

[CR21] Landgrebe M, Azevedo A, Baguley D (2012). Methodological aspects of clinical trials in tinnitus: a proposal for an international standard. J Psychosom Res.

[CR22] Langguth B, Zowe M, Landgrebe M (2006). Transcranial magnetic stimulation for the treatment of tinnitus: a new coil positioning method and first results. Brain Topopr.

[CR24] Leonard CM, Puranik C, Kuldau JM, Lombardino LJ (1998). Normal variation in the frequency and location of human auditory cortex landmarks. Heschl’s gyrus: where is it?. Cereb Cortex.

[CR25] Liegeois-Chauvel C, Musolino A, Chauvel P (1991). Localization of the primary auditory area in man. Brain.

[CR26] Lockwood AH, Salvi RJ, Coad ML, Towsley ML, Wach DS, Murphy BW (1998). The functional neuroanatomy of tinnitus: evidence for limbic system links and neuroplasticity. Neurology.

[CR27] Marcondes RA, Sanchez TG, Kii MA (2010). Repetitive transcranial magnetic stimulation improve tinnitus in normal hearing patients: a double-blind controlled, clinical and neuroimaging outcome study. Eur J Neurol.

[CR29] Mühlnickel W, Elbert T, Taub E, Flor H (1998). Reorganization of auditory cortex in tinnitus. Proc Natl Acad Sci USA.

[CR30] Noh TS, Rah YC, Kyong JS, Kim JS, Park MK, Lee JH, Oh SH, Chung CK, Suh MW (2017). Comparison of treatment outcomes between 10 and 20 EEG electrode location system-guided and neuronavigation-guided repetitive transcranial magnetic stimulation in chronic tinnitus patients and target localization in the Asian brain. Acta Otolaryngol Sep.

[CR31] Norena AJ, Eggermont JJ (2003). Changes in spontaneous neural activity immediately after an acoustic trauma: Implications for neural correlates of tinnitus. Hear Res.

[CR32] Opitz A, Zafar N, Bockermann V, Rohde V, Paulus W (2014). Validating computationally predicted TMS stimulation areas using direct electrical stimulation in patients with brain tumors near precentral regions. Neuroimage Clin Mar.

[CR33] Penhune VB, Zatorre RJ, MacDonald JD, Evans AC (1996). Interhemispheric anatomical differences in human primary auditory cortex: probabilistic mapping and volume measurement from magnetic resonance scans. Cereb Cortex.

[CR34] Plakke B, Romanski LM (2014). Auditory connections and functions of prefrontal cortex. Front Neurosci.

[CR35] Plewnia C, Reimold M, Najib A, Reischl G, Plontke SK, Gerloff C (2007). Moderate therapeutic efficacy of positron emission tomography-navigated repetitive transcranial magnetic stimulation for chronic tinnitus: a randomised, controlled pilot study. J Neurol Neurosurg Psychiatry.

[CR36] Romanski LM, Le Doux JE (1993). Information cascade from primary auditory cortex to the amygdala: corticocortical and corticoamygdaloid projections of temporal cortex in the rat. Cereb Cortex.

[CR37] Romanski LM, Tian B, Fritz J, Mishkin M, Goldman-Rakic PS, Rauschecker JP (1999). Dual streams of auditory afferents target multiple domains in the primate prefrontal cortex. Nat Neurosci.

[CR38] Rossi S, De Capua A, Ulivelli M (2007). Effects of repetitive transcranial magnetic stimulation on chronic tinnitus: a randomised, cross over, double blind, placebo-controlled study. J Neurol Neurosurg Psychiatry.

[CR39] Schecklmann M, Landgrebe M, Poeppl TB (2013). Neural correlates of tinnitus duration and distress: a positron emission tomography study. Hum Brain Mapp.

[CR40] Smith JA, Mennemeier M, Bartel T (2007). Repetitive transcranial magnetic stimulation for tinnitus: a pilot study. Laryngoscope.

[CR41] Theodoroff SM, Folmer RL (2013). Repetitive transcranial magnetic stimulation as a treatment for chronic tinnitus: a critical review. Otol Neurotol.

[CR43] Thielscher A, Opitz A, Windhoff M (2011). Impact of the gyral geometry on the electric field induced by transcranial magnetic stimulation. Neuroimage Jan.

